# CD26/Dipeptidyl Peptidase IV and Its Multiple Biological Functions

**DOI:** 10.7759/cureus.13495

**Published:** 2021-02-22

**Authors:** Kelsey Pan, Kei Ohnuma, Chikao Morimoto, Nam H Dang

**Affiliations:** 1 Internal Medicine, University of Florida, Gainesville, USA; 2 Department of Therapy Development and Innovation for Immune Disorders and Cancers, Juntendo University, Tokyo, JPN; 3 Oncology, University of Florida, Gainesville, USA

**Keywords:** cd26, dipeptidyl peptidase iv, cancer, diabetes, immunology, infectious disease, covid

## Abstract

CD26/Dipeptidyl peptidase IV (DPPIV) is a cell surface glycoprotein with numerous roles including glucose metabolism, immunomodulation, and tumorigenesis. CD26/DPPIV is well recognized in diabetes, with DPPIV inhibitors being a class of oral hypoglycemic drugs called gliptins that are commonly used to treat type two diabetes mellitus. Recent work also indicated a potential role for CD26 in infectious diseases, including COVID-19, and immune-mediated disorders such as rheumatoid arthritis, inflammatory bowel disease, and graft-versus-host disease.

In cancer, CD26/DPPIV expression has been characterized in numerous tumors such as hematologic malignancies, malignant pleural mesothelioma (MPM), renal cell carcinoma (RCC), hepatocellular carcinoma (HCC), gastrointestinal stromal tumor (GIST), and prostate, lung, colorectal, and ovarian (PLCO) cancer. Hence, CD26 has been frequently studied as a tumor biomarker and therapeutic target. CD26/DPPIV-targeted therapies have been evaluated in various cancers, including the use of anti-CD26 monoclonal antibodies as anticancer treatment in selected neoplasms.

This review highlights our current understanding of the role of CD26 in cancer, diabetes, immune-mediated diseases, and infectious diseases. Enhanced understanding of CD26 biology and function may lead to novel therapeutic approaches in multiple human diseases.

## Introduction and background

CD26/Dipeptidyl peptidase IV (DPPIV) is a cell surface glycoprotein that is commonly expressed in many cell types and has numerous biological functions. It cleaves amino-terminal dipeptides with terminal L-proline or L-alanine and is expressed on leukocytes, fibroblasts, mesothelium, endothelial cells, and epithelial cells. It plays a role in multiple biological functions ranging from immunoregulation to glucose homeostasis. Moreover, it is involved in tumorigenesis and may serve as a tumor suppressor or activator, depending on its tumor microenvironment [1-2]. CD26/DPPIV has therefore been extensively studied as a biomarker in various malignancies and as a potential therapeutic target. Interestingly, CD26/DPPIV has recently been implicated to have a role in infectious processes involving Middle East respiratory syndrome coronavirus (MERS-CoV) and also potentially severe acute respiratory syndrome coronavirus 2 (SARS-CoV2) by serving as a cellular receptor to allow for viral entry [2-4]. In this paper, we will review the pertinent literature characterizing the role of CD26/DPPIV, while highlighting some of the major aspects of this molecule in immunology, diabetes, cancer, and infectious diseases.

## Review

CD26 in immune system

CD26/DPPIV in T-Cell Activation

A series of studies demonstrated that CD26/DPPIV has a role in the regulation of the human immune system. A marker of activated T cells, CD26 expression, is upregulated during T-cell activation and is preferentially expressed on CD4+ T memory cells [5]. It is a costimulatory molecule capable of enhancing T lymphocyte activation and proliferation induced through the CD3/T-cell receptor complex as well as the CD2 molecule [6-10]. CD26 involvement in T-cell activation is determined in part by its physical and functional association with a number of key molecules involved in T-cell signal transduction processes, leading eventually to intracellular calcium mobilization, tyrosine phosphorylation of downstream signaling proteins, and increased IL-2 production [11-12]. CD26 also plays a role in human thymocyte activation and thymic differentiation through the CD3 pathway [9].

CD26 in Immune-Mediated Disorders

Having a key role in the signaling processes of T-cell activation, CD26/DPPIV is involved in immune-mediated disorders such as autoimmune diseases and graft-versus-host disease (GVHD). An accumulation of CD26+ lymphocytes was found in target organs involved in GVHD, rheumatoid arthritis (RA), and inflammatory bowel disease (IBD) [13]. CD26 levels have been shown to correlate with disease severity in chronic inflammatory and autoimmune diseases such as RA, IBD, multiple sclerosis, and Graves’ disease, suggesting a role for CD26+ T cells in mediating inflammation and tissue damage. In RA, CD26 levels were inversely correlated with the number of swollen joints. CD26+ T cells are believed to migrate from the peripheral blood into the rheumatoid synovium, thus facilitating inflammation and subsequent tissue destruction in RA. In murine studies, DPPIV inhibitors suppressed RA in a dose-dependent manner [14]. The DPPIV inhibitor sitagliptin was recently found to inhibit fibrosis in systemic sclerosis by inhibiting TGF-B-induced lung fibroblast activation in vitro. It also improved lung injury histologically through the inhibition of proinflammatory cytokines such as IL-1b, TNF-a, and IL-6 [15]. These findings suggest that DPPIV inhibitors may be effective in suppressing immune system in similar inflammatory processes, resulting in clinical improvement of these immune-mediated disorders.

CD26 in Graft-Versus-Host Disease

GVHD is an immune-mediated complication of allogeneic hematopoietic stem-cell transplants (HSCT). Work with a murine model demonstrated that injection of anti-CD26 monoclonal antibodies decreased the severity of GVHD by decreasing IL-26 production, while graft-versus-leukemia effect was still maintained, resulting in prolonged survival [13]. This research suggests that CD26 plays a role in the pathophysiology of GVHD and can be a novel therapeutic target for immune-mediated conditions such as GVHD and chronic inflammatory disorders.

A recent phase II clinical trial showed that treatment with the DPPIV inhibitor sitagliptin in combination with tacrolimus and sirolimus resulted in low incidence of acute GVHD after allogeneic HSCT, compared to 30% in previously published literature. Acute GVHD occurred in two out of 36 patients with an incidence of grade II to IV GVHD of 5%, markedly lower than the observed incidence among patients on sirolimus and tacrolimus alone, which varied from 7% to 47% in prior studies. The one-year cumulative incidence of chronic GVHD was 37% (95% CI: 22% to 53%), compared to observations of 39% to 53% in prior studies [16]. In addition, no toxic effects associated with sitagliptin were observed. This study further supports the role of CD26 in the GVHD, hence the protective properties of DPPIV inhibitors as demonstrated.

CD26 in Diabetes

CD26/DPPIV expression in adipose tissue, pancreatic islet cells, hepatic cells, and microvascular endothelial cells is increased in obesity, diabetes, and other states of inflammation. Incretin hormones such as glucagon-like peptide-1 (GLP-1) and glucose-dependent insulinotropic polypeptide (GIP) regulate post-prandial insulin secretion; however, they are rapidly degraded by CD26/DPPIV. Inhibition of CD26 thus improves post-prandial insulin activity by increasing GLP-1 and GIP levels. Therefore, DPPIV inhibitors, which lower DPPIV activity by 70%-90%, have a significant clinical role in the treatment of type two diabetes [17].

DPPIV-deficient mice have improved glucose tolerance, lower serum glucose levels, and increased insulin secretion after glucose administration [17]. DPPIV inhibition over a longer duration (eight weeks) in mice was associated with increased GLP-1, increased insulin levels, and increased glucose transporter isoform-2 (GLUT-2) expression, even in glucose-intolerant mice. Studies have also suggested the role of DPPIV inhibitors in endothelial growth by inducing endothelial cell proliferation through the activation of TNF-a or IL-1B. These findings suggest a potential role for DPPIV inhibitors in reversing some diabetic vascular complications [17].

In addition, DPPIV levels are elevated in liver diseases, with increased expression in the liver linked to the development of insulin resistance and non-alcoholic fatty liver disease. In mouse models, obesity induces the synthesis of DPPIV by the liver, which subsequently contributes to increased inflammation in adipose tissue, thus exacerbating insulin resistance and metabolic syndrome [18]. Studies with murine models also revealed that DPPIV inhibitors prevented hepatic steatosis and diet-induced inflammation of adipose tissue by inhibiting the infiltration of CD8+ T cells and macrophages [19].

A meta-analysis demonstrated that all DPPIV inhibitors resulted in a moderate reduction of hemoglobin A1c (HbA1c) by 0.5%-0.8%. In a trial of 800 patients with inadequate glycemic control with metformin monotherapy, both saxagliptin and sitagliptin led to reductions in HbA1c by 0.52% and 0.26%, respectively [19]. Amori et al. similarly found that DPPIV inhibitors lowered HbA1c compared to placebo by a weighted mean of 0.74% (95% CI: 0.62%-0.85%). Patients treated with DPPIV inhibitors were found to be more likely to achieve HbA1c less than 7% compared to placebo, without significant differences between sitagliptin and vildagliptin (risk ratio 2.5%; 95% CI: 2.1%-2.8%, p < 0.005) [19]. DPPIV inhibitors were shown to be weight neutral with minimal risk of hypoglycemia and gastrointestinal side effects due to the GLP-1-mediated mechanism of action [18-19].

CD26 in cancer

There is extensive literature evaluating the role of CD26/DPPIV in malignant processes. CD26 expression has been characterized on various cancers such as malignant pleural mesothelioma (MPM), colorectal cancer (CRC), hepatocellular carcinoma (HCC), renal cell carcinoma (RCC), lung cancer, prostate cancer, thyroid cancer, gastrointestinal stromal tumor (GIST), thyroid cancer, and selected hematologic malignancies [20]. CD26 presence has been associated with more aggressive variants in certain cancers through its regulation of metastasis and local invasion, while its absence has also been linked to the development of other cancers due to its ability to regulate cancer progression [1,20]. DPPIV inhibitors are believed to have antineoplastic effects partly by regulating CXCL10-mediated activity of CXCR3+ lymphocyte. CXCR3 is a receptor for CXCL10, and its engagement activates antitumor immune response via the recruitment of T cells, monocytes, and macrophages. CD26/DPPIV cleaves CXCL10 to regulate its biological activity, and DPPIV inhibition prevents degradation of CXCL10, thus increasing CXCR3+ T lymphocyte levels and reducing tumor growth [20]. CD26/DPPIV also increases cell sensitivity to apoptosis in response to topoisomerase II inhibitors, such as doxorubicin and etoposide, in in vitro and in vivo studies [21-23]. Following are some of the key findings regarding the role of CD26 in specific malignancies.

Malignant Mesothelioma

Multiple studies have shown CD26 to be an important tumor marker as well as a novel therapeutic target in malignant mesothelioma. CD26 is expressed at high levels in malignant mesothelioma but not in benign mesothelial cells. A study of 79 patients with MPM showed that 73.4% of MPM exhibited CD26 surface expression. In particular, most epithelioid and biphasic MPM variants, but not sarcomatoid MPM, expressed surface CD26 [24]. CD26 surface expression was also correlated with improved survival in MPM patients who received chemotherapy (mean survival time of 18.6 versus 10.7 months, p = 0.0083), suggesting an association between CD26 expression and mesothelioma chemosensitivity (p = 0.053) [2,24]. Meanwhile, the administration of the humanized anti-CD26 mAb YS110 resulted in growth inhibition of malignant mesothelioma cells in in vitro studies while significantly reducing tumor growth and improving survival in human malignant mesothelioma cell-bearing murine xenograft models. In addition, a first-in-human (FIH) phase I trial involving 33 patients with heavily pretreated CD26-positive malignancies, including 22 patients with malignant mesothelioma, treated with YS110 was recently conducted. Stable disease was observed in half of the patients at 43 days, while prolonged stabilization was seen in seven of 13 patients with stable disease, with a median progression-free survival (PFS) of 33 weeks. This clinical trial demonstrated promising clinical results in CD26-positive MPM patients who previously progressed on multiple chemotherapy agents [25].

In addition, the results of a recently completed phase I trial with Japanese patients with advanced MPM treated with YS110 were reported. Nine Japanese patients were randomized to three different dose cohorts of YS110: two, four, or six mg/kg. While seven of the nine patients developed grade three or four adverse events, most commonly decreased lymphocyte count, and none developed a dose-limiting toxicity. In terms of tumor response, four of seven patients had stable disease, while one achieved a partial response. This phase I trial showed that YS110 administration yielded promising antineoplastic outcome in advanced MPM while remaining relatively well-tolerated, similar to the FIH trial reported previously [26].

Prostate Cancer

Studies of CD26 expression as a biomarker or prognosticator for prostate cancer have demonstrated mixed results. One study showed that malignant prostate tissue expressed twice as high CD26/DPPIV activity compared to benign prostatic hyperplasia (BPH). DPPIV levels were also similarly increased in BPH glands associated with prostatic cancer, suggesting the possible production of local growth factors influencing cancer proliferation. An analysis suggested that high DPPIV expression in prostate cancer tissue samples was associated with poor prognosis (p < 0.0001) [27]. Shah et al. identified 15,330 patients with prostate cancer and type two diabetes through the Surveillance, Epidemiology, and End Results (SEER) and Medicare-linked database and found significantly improved overall survival (OS) in patients on DPPIV inhibitors compared to control (HR 0.77, p = 0.005). Patients with prostate cancer on metformin also had improved OS compared to controls with HR 0.87 (95% CI: 0.81-0.93, p < 0.0001) [28].

On the other hand, an in vitro study by Wesley et al. suggested that DPPIV may have tumor suppressor function by inhibiting the malignant phenotype of prostate cancer via the blocking of basic fibroblast growth factor signaling pathway; therefore, DPPIV inhibition may facilitate tumor growth [29]. Similarly, Sun et al. found that CD26/DPPIV inhibition enhanced invasion and metastasis of prostate cancer cell lines in both in vitro and in vivo assays. CD26/DPPIV cleaves and degrades CXCL12, and in vivo assay found that animals treated with a DPPIV inhibitor had increased prostate cancer cells in all tissues, especially in osseous tissues. These findings suggest that CD26/DPPIV inhibition facilitates prostate cancer invasion into the marrow and metastasis via CXCL12/CXCR4 chemotaxis [30].

Russo et al. found decreased DPPIV mRNA and protein levels in patients with castrate-resistant prostate cancer, and DPPIV inhibition with sitagliptin enhanced prostate cancer xenografts growth after castration. These results suggest that DPPIV may play a role in androgen receptor-regulated tumor suppression, and DPPIV inhibition facilitates growth factor activity and therefore tumor growth [31]. Nazarian et al. found that DPPIV activity was reduced in patients with metastatic prostate cancer compared to those with localized disease or healthy control subjects, though no difference in DPPIV serum levels was noted. Reduced DPPIV activity was shown to be a significant predictor of cancer status after adjusting for age and PSA level [32].

Colorectal Cancer

Numerous studies have suggested the role of CD26 as a biomarker for early CRC detection [33-34]. Cordero et al. reported reduced serum CD26 level in CRC patients compared to healthy controls, especially in the early stages of disease. Serum CD26 levels had a sensitivity of 81.8% for predicting CRC of Dukes’ stages A, B, and C, whereas in stage D, CD26 levels were actually elevated and CEA levels served as a more reliable biomarker [34]. De Chiara et al. measured serum CD26 levels in 299 patients undergoing colonoscopies and found a mean of 641.2 ± 241.2 ng/mL in patients with no colorectal pathology and 403.7 ± 278.2 ng/mL in patients with colorectal cancer. In addition, analysis of polyps revealed a correlation between CD26 levels and grade of dysplasia and the presence of advanced adenomas, with a 58.0% (95% CI: 46.5%-68.9%) sensitivity and 75.5% (95% CI: 68.5%-81.0%) specificity in detecting colorectal cancer and advanced adenomas. Using a 460 ng/mL cut-off, CD26 levels had 81.8% (95% CI: 64.5%-93.0%) sensitivity and 72.3% (95% CI: 65.0%-77.2%) specificity in predicting the absence of or benign colorectal pathology [34].

While prior studies have mostly identified lower serum CD26 levels in CRC, Lam et al. analyzed tumor CD26 expression levels in CRC patients and found significantly higher levels in those with distant metastases compared to non-metastatic disease. In addition, patients with high CD26 expression were found to have worse OS (p < 0.0001). Larrinaga et al. similarly measured higher DPPIV activity and mRNA expression in tissue samples of CRC and colon adenomas compared to non-neoplastic tissues while noting significantly lower plasma DPPIV activity in CRC patients [35]. DPPIV inhibition with vidagliptin suppressed the incidence and growth of lung metastases in CRC in mice through increased cell apoptosis by downregulating autophagy and cell cycle modulation. Following treatment with vildagliptin, a decrease in autophagy markers such as LC3, p62, and ATF4; an increase in apoptosis; and inhibition of cell cycle regulator pCDC2 were observed [36].

CD26 has been implicated as a marker of cancer stem cell in CRC by various studies. Injection of isolated CD26+ cells from patients with metastatic CRC into the cecal walls of mice led to the development of distant metastasis, but injection of CD26 negative (CD26-) cells did not. Hence, a subpopulation of CD26+ cancer cells was thought to have stem-like features and were also preferentially found in metastatic liver tissues. In the study, CD26+ cells were found in all primary and metastatic tumors in 20 patients with liver metastasis and in only eight of 27 patients without liver metastasis [37]. Furthermore, Lam et al. demonstrated that higher levels of CD26 expression detected by immunohistochemistry correlated with more advanced tumor stage and worse survival rate [38]. A study by Cheung et al. quantified CD26+ cancer cells in 11 primary CRC tissue samples and showed metastatic tumors to have relatively high CD26+ levels. Specifically, the CD26+ proportion in CRC tumors with metastasis was 7.20% ± 5.20% and 0.43% ± 0.15% in those without metastasis (p = 0.13). Tissues with higher CD26+ levels correlated with the presence of metastases (p = 0.0061) and even led to the later diagnosis of metastatic disease in two subjects who did not initially have metastases at the time of study enrollment. It was hypothesized that the subpopulation of cancer stem cells arises from CD26- daughter cells via gene manipulation of PIK3CA and TP53 during later stages of carcinogenesis [39].

Lung Cancer

Contrary to certain other cancers, CD26 was actually detected at a reduced or undetectable level in most non-small cell lung cancers (NSCLC) compared to normal bronchial and alveolar epithelial cells. However, CD26 expression was increased only in lung adenocarcinoma, making it a potential tool for distinguishing between lung cancer subtypes [36,40]. Asada et al. examined DPPIV enzyme activity in numerous histological types of lung carcinomas and found that 93.1% of lung adenocarcinoma tissues expressed positive staining for DPPIV activity, while all cases of squamous cell, small cell, carcinoid, and large cell carcinoma were negative. CD26-expressing cells in non-adenocarcinoma lung cancers contained an increased proportion of cells in G0-G1 stages, suggesting a role for CD26 in promoting cell cycle arrest [40]. Wesley et al. similarly found absent or markedly reduced DPPIV expression in all NSCLC cells at both mRNA and protein levels, while normal lung epithelial cells had detectable DPPIV expression. Interestingly, restoring DPPIV expression in NSCLC cells led to inhibition of cell proliferation, anchorage-independent growth, in vitro cell migration, tumorigenicity, increased p21 expression, and therefore apoptosis and cell cycle arrest [29]. Hence, DPPIV is linked to suppression of tumor growth and metastasis of NSCLC, and its loss of function is believed to contribute to the development of NSCLC.

As significantly higher DPPIV expression was found in lung adenocarcinoma, Jang et al. evaluated the effects of DPPIV inhibition by vidagliptin on tumor growth in lung adenocarcinoma. The study showed that vidagliptin reduced growth of lung adenocarcinoma through activation of natural killer cell activity and surfactant-activated macrophages [36]. Bishnoi et al. demonstrated that DPPIV inhibitors resulted in a survival advantage in diabetic patients with lung cancer or colorectal cancer (HR 0.89; 95% CI: 0.82-0.97, p = 0.0007), with a synergistic survival advantage when used in conjunction with metformin (HR 0.83; 95% CI: 0.77-0.90, p < 0.0001) [41].

Hematologic Malignancies

CD26 expression has been associated with aggressive T-large granular lymphocyte (T-LGL) lymphoproliferative disorder, with patients having CD26+ disease being more likely to acquire infections and cytopenias requiring treatment than those with CD26 negative disease [42]. Carbone et al. examined 67 human T-cell Non-Hodgkin’s lymphomas and leukemias and found that CD26 expression was exclusive to aggressive pathologies, such as T-cell lymphoblastic lymphoma (LBL)/T-cell acute lymphoblastic leukemia (ALL) and T-cell CD30+ anaplastic large cell (ALC) lymphomas. In addition, CD26 expression has also been associated with higher degree of disease aggressiveness and worse survival outcomes in T-LBL/T-ALL compared to patients with CD26 negative tumors (p < 0.0001). On the other hand, CD26 was undetectable in most tissue samples of mycosis fungoides/Sézary syndrome subtype of T-cell lymphoma. Therefore, CD26 has a potential role as a biomarker for distinguishing among subtypes of T-cell malignancy [43-44].

Reinhold et al. used two CD26/DPPIV inhibitors to suppress DPPIV function of human histiocytic lymphoma cells, which resulted in suppressed DNA synthesis and cytokine production in those with high DPPIV expression. These findings support the hypothesis that CD26/DPPIV contributes to the growth of T-cell lymphoma through cytokine production [45]. Ho et al. demonstrated that administration of anti-CD26 monoclonal antibody resulted in cell cycle arrest and inhibition of tumor proliferation of human CD30+ ALC T-cell lymphoma, suggesting potential clinical benefit of anti-CD26 antibody therapy in CD26+ hematological malignancies as well as potentially CD26+ cancers in general [46].

CD26 in infectious diseases

CD26 in MERS

CD26/DPPIV has been found to play a role in infection mediated by Middle East respiratory syndrome coronavirus (MERS-CoV) and, more recently, potentially by severe acute respiratory syndrome coronavirus 2 (SARS-CoV2) also known as COVID-19. MERS-CoV emerged in 2012 in the Middle East, leading to fatal lower respiratory tract infections with a 35% fatality rate [3]. CD26 is expressed in numerous cell types including bronchial mucosa, alveolar cells (in particular, type two alveolar cells), T lymphocytes, and the systemic circulation. It also functions as a receptor by which the spike protein S1, which is a type I transmembrane glycoprotein, of MERS-CoV is allowed entry into human cells [20]. MERS-CoV activates an inflammatory response by binding to S-protein, which causes a conformational change and interactions with T lymphocytes [4]. Due to the newfound involvement of CD26 in MERS-CoV infectivity, CD26 inhibition raises interest as a potential therapeutic target for the treatment of MERS-CoV.

While CD26/ DPPIV chemical inhibitors did not inhibit binding between DPPIV and MERS-CoV, the use of antibodies directed against CD26 has shown more promising results in MERS-CoV-mediated infection [20]. In a mouse study by Li et al., human DPP4 knockin mice with humanized exons of the mouse DPP4 locus were inoculated with MERS-CoV. Viral replication was observed in the lungs, although these mice did not develop illness [4]. Ohnuma et al. demonstrated that treatment with anti-CD26 mAbs successfully inhibited interaction between CD26 and the spike protein, therefore suppressing MERS-CoV host infection. Various clones of anti-CD26 mAbs and the humanized anti-CD26 mAb YS110 were tested in binding inhibition assays with the fusion protein construct MERS-CoV S1-Fc, with the anti-CD26 mAbs 2F9, 1F7, and YS110 being able to block MERS-CoV infection in vitro [21]. In addition, the humanized anti-CD26 mAb YS110 exhibited tolerable safety profiles in a phase I clinical trial involving patients with CD26-expressing malignancies, as discussed earlier [20,25]. These findings suggest the potential of anti-CD26 mAbs as a novel therapeutic approach in MERS-CoV-mediated infection.

CD26 in COVID-19

The SARS-CoV-2, also known as COVID-19, pandemic has affected over 3.8 million individuals in over 200 countries in 2020. The disease is characterized by severe acute respiratory syndrome with a case fatality rate of 4.58% [47]. Recent work has shown an association between COVID-19 case fatality and hypertension and type two diabetes [47-49]. Coronaviruses have been demonstrated to gain entry into human cells through the spike protein (S-protein) that interacts with certain membrane receptors. In the case of SARS-CoV-2, virus gains cell entry through a type II transmembrane serine protease called TMPRSS2, which activates the viral spike protein and allows binding to angiotensin-converting enzyme two (ACE2). TMPSS2 and ACE2 are co-expressed by type II pneumocytes of human lungs [49]. DPPIV may also function as a co-receptor for viral entry of SARS-CoV-2 by facilitating S-protein binding [47-48]. Computational models have suggested an association between DPPIV dysfunction and COVID-19 disease severity based on the anti-inflammatory effects of DPPIV inhibitors such as vildagliptin and saxagliptin shown in animal studies [48]. Further experimental studies are needed to evaluate the clinical impact of DPPIV inhibition on COVID-19 disease course.

A recent genetic study by Senapati et al. on the interactions among SARS-CoV-2 spike protein, TMPRSS2, and CD26 further characterized the potential role of CD26 in COVID19 infection. CD26 is found to be significantly involved in the expression of key regulatory genes that regulate SARS-CoV-2 internalization. Furthermore, epigenetic modifications that induce CD26 overexpression are hypothesized to have a role in the higher case fatality rate of SARS-CoV-2 among type two diabetics; however, results have not been proven in experimental studies [47]. Due to the known anti-inflammatory effects of DPPIV inhibitors by reducing C-reactive protein and IL-6 levels, it has been hypothesized that DPPIV inhibitors such as sitagliptin may play a role in reducing inflammation in SARS-CoV-2 infection [48]. However, there is currently no published experimental data showing that DPPIV inhibitors decrease binding and viral entry.

## Conclusions

The diverse role of CD26/DPPIV in immunology, tumorigenesis, glucose homeostasis, and infectious pathophysiology has been established in numerous studies over the past decades. Beyond the pathophysiological involvement of CD26 in various diseases, recent in vivo studies and clinical trials suggest it to be a promising therapeutic target. For example, DPPIV inhibitor sitagliptin reducing the rate of acute GVHD following allogeneic HSCT suggests an important role in immunosuppression and clinical outcomes of immune-mediated disorders. Furthermore, the administration of the humanized anti-CD26 mAb YS110 yielded promising antineoplastic outcomes in advanced MPM that previously progressed on numerous chemotherapy agents, suggesting a more well-tolerated and possibly more effective treatment potential in certain cancers.

In the arena of infectious disease, studies of mice injected with anti-CD26 mAbs demonstrated successful suppression of MERS-CoV host infection, although this was not tested in human studies prior to the eradication of MERS. More relevant to current times, CD26 is believed to function as a co-receptor for viral entry of COVID-19, while DPPIV inhibitors are hypothesized to reduce the inflammatory response. More experimental and clinical data are needed to demonstrate the role of DPPIV inhibitors in reducing viral entry or downstream inflammation, but CD26/DPPIV remains a promising therapeutic target.
